# Prevalence and trends in recent alcohol consumption by cancer history and cancer type in a nationally representative sample of U.S. adults

**DOI:** 10.1007/s10552-026-02248-8

**Published:** 2026-09-24

**Authors:** Kimberly J. Johnson, Amy A. Eyler, Jenine K. Harris

**Affiliations:** 1 Brown School, Washington University in St. Louis, Goldfarb Hall, Room 237, Campus Box 1196, One Brookings Drive, St. Louis, MO 63130 USA; 2 Bursky School of Public Health, Washington University in St. Louis, St. Louis, MO USA; 3 Office of the Provost, Washington University in St. Louis, St. Louis, MO USA

**Keywords:** Alcohol, Cancer, Trends, National, Survey

## Abstract

**Purpose:**

Alcohol consumption increases the risk of several cancer types. Using nationally representative data, we aimed to 1) quantify trends in alcohol consumption by cancer history and 2) estimate prevalence differences in alcohol consumption comparing individuals with vs. without a cancer history overall and by cancer type.

**Methods:**

We used Behavioral Risk Factor Surveillance System (BRFSS) cross-sectional data from 2016–2024 for trend and 2022–2024 for prevalence difference analyses. We calculated weighted prevalences of current (past-30-day alcohol consumption), binge, and heavy drinking among individuals with and without a cancer history. Annual percentage-point changes (APPCs) and adjusted prevalence differences were estimated using linear regression.

**Results:**

Data from 3,968,799 and 465,870 respondents were included in trend and prevalence difference analyses. Across all years, the current drinking prevalence was lower among individuals with a cancer history than without. Current drinking declined among those without a cancer history (53.9% in 2016 to 51.5% in 2024; APPC = − 0.28%, *p*<.05) but not among those with a cancer history (45.8% in both 2016 and 2024; APPC = 0.06%, *p*=.70). The adjusted prevalence of current and binge drinking was lower in those with a cancer history. By cancer type, the adjusted prevalence of current drinking was lower for most sites except for testicular, skin, and melanoma.

**Conclusions:**

Alcohol consumption was generally less prevalent among those with vs. without a reported cancer history and modestly declined over time in the latter group. These results inform the understanding of recent patterns in alcohol consumption by cancer history.

**Supplementary Information:**

The online version contains supplementary material available at https://doi.org/10.1007/s10552-026-02248-8.

## Introduction

Cancer is the second leading cause of U.S. deaths, with approximately two million new diagnoses and over 600,000 deaths annually [1]. A growing body of evidence indicates that a proportion of cases may be preventable, with meta-analyses over the past decade confirming that alcohol consumption increases the risk for several cancer types [2–4]. According to the U.S. National Cancer Institute (NCI), alcohol is associated with increased risks of head and neck, esophageal, liver, breast, and colorectal cancers. The NCI also notes some evidence for elevated risks of melanoma, pancreatic, prostate, and stomach cancers, while associations with ovarian, uterine, and bladder cancers are inconsistent. Reduced risks have been reported for kidney, thyroid, and non-Hodgkin lymphoma [5]. Importantly, alcohol consumption may also increase the risk of second primary (new) cancers among cancer survivors [6, 7].

Although some studies have shown that individuals reduce their alcohol intake after a cancer diagnosis—for example, colorectal cancer survivors decreased consumption in the two years following diagnosis [8]—alcohol use remains common. Health Information National Trends Survey (HINTS) data from 2000–2017 indicated that 56.5% of cancer survivors reported drinking alcohol in the past year, including 21% who were classified as binge drinkers [9]. Similarly, National Health and Nutrition Examination Survey (NHANES) data from 1999 to 2016 showed that although cancer survivors were less likely than individuals without a cancer history to report current drinking, a substantial proportion still consumed alcohol [10].

Despite increasing scientific consensus on the carcinogenic effects of alcohol, public awareness remains low. Over the past decade, there have been growing calls for public health interventions, including warning labels on alcoholic beverages [11]. In 2019, consumer and public health groups advocated for labeling, and in 2025, the U.S. Surgeon General echoed this call [12, 13]. Public interest in the alcohol–cancer link has also grown, with Google Trends data normalized to total searches showing an upward trajectory for searches related to alcohol and cancer risk since 2004 [14]. Yet, as of 2024, fewer than half of U.S. adults (39.4%) were aware that alcohol increases cancer risk [15].

While there may be a growing public discourse around alcohol and cancer [16], empirical evidence for population-level changes in alcohol use is limited, particularly among those with a cancer history. Most prior studies have been limited to single time points, and recent alcohol consumption trend data in cancer survivors vs. individuals without a cancer history are lacking. Moreover, little is known about whether survivors of alcohol-related cancers, such as breast and colorectal cancer, report lower alcohol use than survivors of cancers not alcohol-associated. Although alcohol use screening is recommended in oncology and primary care settings, recent research indicates that heavy drinkers with a cancer history are not consistently counseled to reduce consumption [17].

In this study, we had two objectives. First, we examined trends in alcohol consumption from 2016 to 2024 among individuals with and without a reported cancer history. Examining trends in alcohol consumption provides a contemporary baseline against which future changes in alcohol consumption among the general population without a past cancer diagnosis and survivors can be assessed, as recommendations about consumption [18] and the alcohol environment evolve [19]. We hypothesized that alcohol use has declined over time, particularly among cancer survivors, as a result of media reports over the last several years [20, 21]. Second, we estimated adjusted differences in the prevalence of alcohol consumption overall, by alcohol-associated cancer type, and by individual cancer types, hypothesizing that individuals with a history of an alcohol-related cancer would report lower levels of alcohol use than those with no cancer history and cancers not strongly associated with alcohol. These findings may help inform targeted health communication and survivorship care strategies aimed at reducing alcohol-related cancer risks.

## Materials & methods

### Study population

We downloaded Behavioral Risk Factor Surveillance System (BRFSS) data for each year from 2016 to 2024 from the U.S. Centers for Disease Control and Prevention (CDC) website [22]. BRFSS is a telephone survey system that collects data from more than 400,000 U.S. residents across all 50 states, the District of Columbia, and U.S. territories on health behaviors, medical conditions, and the use of preventive services, such as cancer screening [23]. The BRFSS questionnaire includes a core component and optional modules added by states/territories [24]. Inclusion and exclusion criteria were based on available data. For *trend analyses* evaluating changes in alcohol consumption over time, we included individuals participating in BRFSS surveys from 2016 to 2024 in all 50 states, the District of Columbia, Puerto Rico, Guam, and the Virgin Islands who answered alcohol consumption questions as part of the core module. Of note, survey data were missing for FL in 2021, KY and PA in 2023, NJ in 2019, the Virgin Islands in 2017 to 2020, and TN in 2024. Therefore, these states/territories were excluded from prevalence calculations for these years. For our analyses of adjusted prevalence differences between alcohol consumption and a reported cancer history, we used the most recent BRFSS data from 2022 to 2024, which may be more relevant to current population patterns. These data were from the 38 states/territories that included the optional module “Cancer Survivorship: Type of Cancer” (See Supplementary Table 1) [25, 26]. The study utilized de-identified publicly available data from the BRFSS and is exempt from Washington University Institutional Review Board approval.

### Variables

We harmonized variables with slight year-to-year measurement variation before imputation (See Supplementary Table 2 for a description of the BRFSS variables that were used and how they were harmonized). Briefly, we used “ALCDAY4” and “ALCDAY5” that asked: “During the past 30 days how many days per month did you have at least one drink of any alcoholic beverage…?” to categorize individuals as current drinkers or non-drinkers within the past 30 days. Individuals with a value <300 (at least 1 drink per week or month) and 888 (No drinks in the past 30 days) were categorized as having and not having an alcoholic drink in the last 30 days, respectively, with all other responses coded as missing before imputation (see below). The derived variables "_RFBING5" and "_RFDRHV#", with the final digit varying by year for "_RFDRHV#", were used to classify individuals as binge and heavy drinkers. Binge drinkers were defined by BRFSS as males “having five or more drinks on one occasion” and females “having four or more drinks on one occasion”. Heavy drinkers were defined by BRFSS as “adult men having more than 14 drinks per week and adult women having more than 7 drinks per week” [27].

Cancer history was ascertained through the variables “CHCOCNC1” (years 2022 to 2024) and “CHCOCNCR” (years 2016 to 2021) that asked “(Ever told) (you had) melanoma or any other types of cancer?” and “(Ever told) you had any other types of cancer?” respectively. Of note, from 2016 to 2021, the question asked about *other* cancer types (the prior survey question asked about skin cancer in general). Therefore, melanoma was not ascertained in the cancer history question for the years 2016 to 2021 but was included in 2022 to 2024. Those who responded yes to either of these questions were coded as having a cancer history, while those who responded no were coded as not having a cancer history, with all others coded as missing before imputation (described below). To define cancer types, we used the variable “CNCRTYP2” in the 2022, 2023, and 2024 data, asking “What kind of cancer is it?”. Although non-melanoma skin cancers are often excluded, we included this cancer type in our analyses for comparison purposes as there have been reports of positive associations between alcohol consumption and these types of cancers [28]. Other analytic variables included sex, race/ethnicity, current smoking, health insurance, age, personal healthcare provider, and income category (see Supplementary Table 2 for coding). We further classified cancer types into alcohol-related vs. not alcohol-related/uncertain. Alcohol-related cancers were defined according to the NCI as head and neck cancers (excluding those of the lips), esophageal cancer, liver cancer, breast cancer, and colorectal cancer [5], with all other reported cancer types being classified as not alcohol related/uncertain. Of note, we included lip cancer as an alcohol-related cancer, as it was not possible to separate it from mouth/tongue/lip, and the case number was small. Primary sampling unit (PSU), weight, and strata were identified by the “PSU”, “_LLCPWT”, “_LLCPWT2”, and “_STSTR” variables.

### Statistical analysis

#### Imputation

For the trend analysis using 2016 to 2024 data and the prevalence difference analysis using 2022 to 2024 data, we conducted three and five imputations, respectively, using the mice function from the R mice package (see Supplementary Materials & Methods for further details).

#### Weighted estimates 

For the trend analysis, we used the R *survey* package svydesign() function to account for the complex sampling design, specifying the primary sampling unit (_PSU), the BRFSS sampling stratification variable (_STSTR), and the sampling weight (_LLCPWT). BRFSS uses stratified sampling within state-based telephone sampling frames, including disproportionate stratified sampling for landline samples and random sampling for cellular telephone samples [27]. Weighted prevalences and corresponding design-based variances were calculated for each imputed dataset and pooled using Rubin’s rules [29]. Variance estimation for strata containing a single PSU used the R option *survey.lonely.psu* = *"adjust".* For analyses using 2022 to 2024 data, sampling weights for each year were multiplied by the proportion of observations contributed by that year to the pooled dataset as described in BRFSS documentation [30]. Accordingly, 2022, 2023, and 2024 weights were multiplied by 0.44, 0.28, and 0.28, respectively.

#### Descriptive and analytic analyses

For the trend analysis, linear trends in the pooled annual prevalence estimates were assessed using ordinary least squares regression of prevalence on calendar year, with the regression coefficient representing the annual percentage-point change (APPC). For prevalence difference analyses, survey-weighted linear regression models using the *svyglm()* function with a Gaussian family were fit separately within each imputed dataset using the specified BRFSS survey design. Unadjusted and covariate-adjusted predicted alcohol prevalences and prevalence differences (PDs) between respondents with and without a reported cancer history were then estimated within each imputed dataset. Design-based variances were estimated using Taylor series linearization accounting for the BRFSS sampling strata and primary sampling units [31, 32]. Estimates and corresponding variances were subsequently pooled across imputations using Rubin’s rules [29]. Cancer type-specific models focused on current drinking as the outcome because small cell sizes for many cancer types and the low prevalence of binge and heavy drinking limited stable estimation. For breast, cervical, uterine, and ovarian cancers, only females were included in models, and for testicular and prostate cancers, only males were included in models. We also conducted sensitivity analyses excluding individuals who reported being in current treatment. All prevalence difference models were adjusted for age category, sex, race/ethnicity, education, insurance, current smoking, marital status, personal healthcare provider, and income category, confounders that were selected based on a directed acyclic graph (https://dagitty.net/mbvsWNRaM). We set alpha at 0.05 and considered p-values significant at *p* < 0.05. R version 4.5.0 was used for all analyses.

## Results

### Analytic dataset description

The total number of respondents in each imputed dataset was 3,968,799 from 2016 to 2024 (trend analysis) and 465,870 from 2022 to 2024 (prevalence difference analyses). A total of 1,038,206 (26.2%) and 136,904 (29.4%) observations had at least one missing value among variables included in multiple imputation models for each study period’s data, respectively. Among the questions asked of all respondents, income had the highest missing data percentage at 19.1 and 20.9%, respectively (See Supplementary Table 3).

### Characteristics of the study participants

Individuals with a reported cancer history from 2016 to 2024 were more likely to be ≥ 65 years (66.7% vs. 32.5%), be Non-Hispanic (NH) White (85.9% vs. 74.3%), be female (59.7% vs. 53.6%), have less than a high school education (40.6% vs. 39.5%), be married (52.7% vs. 51.6%), have incomes > $50,000 per year (51.6% vs. 47.4%), have health insurance (97.6% vs. 92.4%), and have a personal healthcare provider (95.1% vs. 84.1%) than those without a cancer history. They were less likely to be current smokers (11.7% vs. 13.6%), current drinkers (45.9% vs. 52.4%), binge drinkers (7.0% vs. 14.4%), and heavy drinkers (5.0% vs. 6.2%) than those without a cancer history. Pattern directions were similar in the 2022 to 2024 data (Table 1). The most common specific cancer types in the 2022 to 2024 data were breast, skin (non-melanoma), melanoma, and prostate cancer (Supplementary Table 4).

**Table 1:** Unweighted characteristics of the study population^a^

	2016 to 2024 trend analyses			2022 to 2024 association analyses		
	No reported cancer (*n *= 3,554,919)	Reported cancer (*n *= 413,880)	Overall (*n *= 3,968,799)	No reported cancer (*n *= 412,343)	Reported cancer (*n*= 53,527)	Overall (*n *=465,870)
Age category						
18 to 24	236,962 (6.7%)	1,619 (0.4%)	238,581 (6.0%)	28,676 (7.0%)	200 (0.4%)	28,876 (6.2%)
25 to 34	415,281 (11.7%)	6,670 (1.6%)	421,951 (10.6%)	49,001 (11.9%)	658 (1.2%)	49,659 (10.7%)
35 to 44	480,028 (13.5%)	15,429 (3.7%)	495,457 (12.5%)	59,726 (14.5%)	1,886 (3.5%)	61,612 (13.2%)
45 to 54	563,999 (15.9%)	35,314 (8.5%)	599,313 (15.1%)	64,571 (15.7%)	4,339 (8.1%)	68,910 (14.8%)
55 to 64	701,637 (19.7%)	78,742 (19.0%)	780,379 (19.7%)	75,971 (18.4%)	9,792 (18.3%)	85,763 (18.4%)
65+	1,157,012 (32.5%)	276,106 (66.7%)	1,433,118 (36.1%)	134,398 (32.6%)	36,652 (68.5%)	171,050 (36.7%)
Race/ethnicity category						
NH White	2,639,588 (74.3%)	355,558 (85.9%)	2,995,146 (75.5%)	301,733 (73.2%)	47,137 (88.1%)	348,870 (74.9%)
NH Black	291,158 (8.2%)	22,210 (5.4%)	313,368 (7.9%)	37,051 (9.0%)	2,587 (4.8%)	39,638 (8.5%)
NH American Indian/Alaskan Native	60,061 (1.7%)	5,568 (1.3%)	65,629 (1.7%)	5,844 (1.4%)	536 (1.0%)	6,380 (1.4%)
NH ASIAN	96,885 (2.7%)	3,535 (0.9%)	100,420 (2.5%)	12,019 (2.9%)	380 (0.7%)	12,399 (2.7%)
NH Asian Pacific Islander (API)	17,265 (0.5%)	947 (0.2%)	18,212 (0.5%)	ND	ND	ND
NH API/MULTI/OTHER	101,921 (2.9%)	9,868 (2.4%)	111,789 (2.8%)	12,046 (2.9%)	1,076 (2.0%)	13,122 (2.8%)
HISPANIC	348,041 (9.8%)	16,194 (3.9%)	364,235 (9.2%)	43,650 (10.6%)	1,811 (3.4%)	45,461 (9.8%)
Sex						
Male	1,648,558 (46.4%)	166,984 (40.3%)	1,815,542 (45.7%)	195,339 (47.4%)	23,536 (44.0%)	218,875 (47.0%)
Female	1,906,361 (53.6%)	246,896 (59.7%)	2,153,257 (54.3%)	217,004 (52.6%)	29,991 (56.0%)	246,995 (53.0%)
Education level						
< High school	1,403,394 (39.5%)	168,035 (40.6%)	1,571,429 (39.6%)	174,657 (42.4%)	23,985 (44.8%)	198,642 (42.6%)
High school	969,665 (27.3%)	117,184 (28.3%)	1,086,849 (27.4%)	108,446 (26.3%)	14,804 (27.7%)	123,250 (26.5%)
Some college	940,909 (26.5%)	104,305 (25.2%)	1,045,214 (26.3%)	104,637 (25.4%)	12,394 (23.2%)	117,031 (25.1%)
College or more	240,951 (6.8%)	24,356 (5.9%)	265,307 (6.7%)	24,603 (6.0%)	2344 (4.4%)	26,947 (5.8%)
Marital status						
Married	1,836,092 (51.6%)	217,930 (52.7%)	2,054,022 (51.8%)	211,300 (51.2%)	29,333 (54.8%)	240,633 (51.7%)
Unmarried	1,718,827 (48.4%)	195,950 (47.3%)	1,914,777 (48.2%)	201,043 (48.8%)	24,194 (45.2%)	225,237 (48.3%)
Income						
<50 K	1,871,238 (52.6%)	200,148 (48.4%)	2,071,386 (52.2%)	242,051 (58.7%)	30,401 (56.8%)	272,452 (58.5%)
50 K+	1,683,681 (47.4%)	213,732 (51.6%)	1,897,413 (47.8%)	170,292 (41.3%)	23,126 (43.2%)	193,418 (41.5%)
Health insurance						
No insurance	268,721 (7.6%)	9,895 (2.4%)	278,616 (7.0%)	24,641 (6.0%)	732 (1.4%)	25,373 (5.4%)
Insurance	3,286,198 (92.4%)	403,985 (97.6%)	3,690,183 (93.0%)	387,702 (94.0%)	52,795 (98.6%)	440,497 (94.6%)
Personal healthcare provider						
No personal doctor	564,586 (15.9%)	20,155 (4.9%)	584,741 (14.7%)	54,105 (13.1%)	1,842 (3.4%)	55,947 (12.0%)
Personal doctor	2,990,333 (84.1%)	393,725 (95.1%)	3,384,058 (85.3%)	358,238 (86.9%)	51,685 (96.6%)	409,923 (88.0%)
Current smoker						
Non-smoker	3,073,031 (86.4%)	365,632 (88.3%)	3,438,663 (86.6%)	363,230 (88.1%)	48,109 (89.9%)	411,339 (88.3%)
Current smoker	481,888 (13.6%)	48,248 (11.7%)	530,136 (13.4%)	49,113 (11.9%)	5,418 (10.1%)	54,531 (11.7%)
Current drinker						
No drinks	1,691,792 (47.6%)	224,012 (54.1%)	1,915,804 (48.3%)	196,552 (47.7%)	27,828 (52.0%)	224,380 (48.2%)
Any drink in the last 30 days	1,863,127 (52.4%)	189,868 (45.9%)	2,052,995 (51.7%)	215,791 (52.3%)	25,699 (48.0%)	241,490 (51.8%)
Binge drinker						
Not binge drinker	3,043,109 (85.6%)	384,941 (93.0%)	3,428,050 (86.4%)	352,566 (85.5%)	49,537 (92.5%)	402,103 (86.3%)
Binge drinker	511,810 (14.4%)	28,939 (7.0%)	540,749 (13.6%)	59,777 (14.5%)	3,990 (7.5%)	63,767 (13.7%)
Heavy drinker						
Not heavy drinker	3,333,773 (93.8%)	393,290 (95.0%)	3,727,063 (93.9%)	387,580 (94.0%)	50,878 (95.1%)	438,458 (94.1%)
Heavy drinker	221,146 (6.2%)	20,590 (5.0%)	241,736 (6.1%)	24,763 (6.0%)	2,649 (4.9%)	27,412 (5.9%)

^a^From the first imputation dataset

*NA* Not applicable, *ND* Not determined

### Trend analyses

**Fig. 1:**
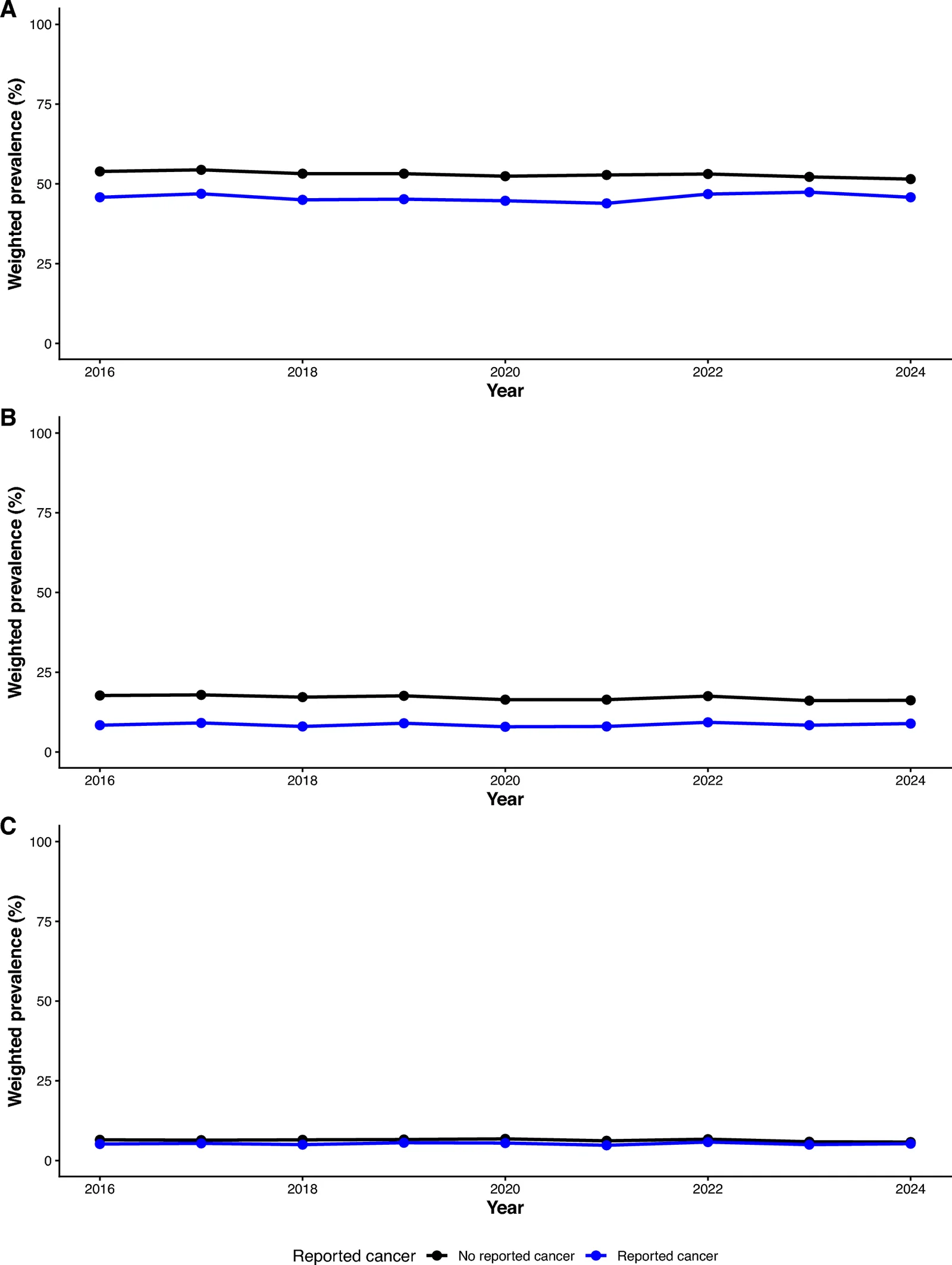
Weighted prevalences by reported cancer history of **A** current drinking, **B** heavy drinking, and **C** binge drinking

The weighted prevalence of current drinking among those with vs. without a reported cancer history was consistently lower than in those without cancer history from 2016 to 2024 (Fig. 1A). We found a non-significant increase in alcohol consumption of 0.06 percentage points per year (95% CI –0.31 to 0.43%, *p* = 0.70) in those with a cancer history and a significant decrease of 0.28 percentage points per year (95% CI − 0.42 to − 0.14%, *p* = 0.002) in those without a cancer history. A similar pattern was found for binge and heavy drinking, with consistently lower weighted prevalences across the study period in those with vs. without a reported cancer history (Fig. 1B and C). Binge drinking non-significantly increased by an average of 0.03 (95% CI − 0.15 to 0.20) percentage points per year (*p* = 0.74) in those with a cancer history and significantly declined by an average of − 0.20 (95% CI − 0.35 to − 0.05) percentage points per year (*p* = 0.02) in those without a cancer history. No significant trends were found for heavy drinking in either group during the study period. Unweighted prevalences, weighted prevalences, and regression results for APPC estimation are provided in Supplementary Fig. 1, Supplementary Tables 5 and 6.

### Weighted adjusted prevalence differences for cancer overall, alcohol-related cancers, and specific cancer types

The adjusted prevalence of current drinking, binge drinking, and heavy drinking was 1.01% lower (aPD= − 1.01; 95% CI − 1.85 to − 0.16), 1.16% lower (aPD = − 1.16; 95% CI − 1.68 to − 0.65), and 0.12% higher (aPD = 0.12; 95% CI − 0.28 to 0.52) in those with than without a reported cancer history. Those with alcohol-related cancers had a significantly lower adjusted prevalence of current drinking (aPD = − 2.17; 95% CI − 3.86 to − 0.48) but not binge drinking (aPD = − 0.34 95% CI − 1.20 to 0.53) nor heavy drinking (aPD = 0.10; 95% CI − 0.59 to 0.80) (Table 2). We also evaluated patterns in alcohol consumption in those who were 55 years and older. Patterns were generally similar in direction for most categories except heavy drinking; however, these results were not statistically significant (Table 2).

**Table 2:** Weighted associations between current drinking and reported cancer history, overall and in those 55 years and older

	Any drink within last 30 days		Binge drinking		Heavy drinking	
	PD (95%CI)^a^	aPD (95%CI)^b^	PD (95%CI)^a^	aPD (95%CI)^b^	PD (95%CI)^a^	aPD (95%CI)^b^
18 years and older (*n* = 465,870)						
Cancer history						
No	0.0 (reference)	0.0 (reference)	0.0 (reference)	0.0 (reference)	0.0 (reference)	0.0 (reference)
Yes	−**5.38 (**−** 6.25 to **−** 4.51)**	−**1.01 (**−** 1.85 to − 0.16)**	−**7.64 (**−** 8.16 to **−** 7.11)**	−**1.16 (**− **1.68 to − 0.65)**	−**0.74 (**−** 1.14 to − 0.35)**	0.12 (− 0.28 to 0.52)
Alcohol-related cancer						
No reported cancer	0.0 (reference)	0.0 (reference)	0.0 (reference)	0.0 (reference)	0.0 (reference)	0.0 (reference)
No/Uncertain	−**4.06 (**−** 5.02 to **−** 3.10)**	−0.70 (− 1.62 to 0.22)	−**7.14 (**−** 7.73 to **−** 6.55)**	−**1.38 (**− **1.97 to **−** 0.80)**	−**0.66 (**−** 1.12 to **−** 0.20)**	0.13 (− 0.33 to 0.59)
Yes	−**10.34 (**−** 12.10 to **−** 8.58)**	−**2.17 (**−** 3.86 to **−** 0.48)**	−**9.52 (**−** 10.43 to **−** 8.60)**	−0.34 (− 1.20 to 0.53)	−**1.05 (**−** 1.73 to **−** 0.37)**	0.10 (− 0.59 to 0.80)
55 years and older (*n* = 256,813)						
Cancer history						
No	0.0 (reference)	0.0 (reference)	0.0 (reference)	0.0 (reference)	0.0 (reference)	0.0 (reference)
Yes	−**0.99 (**−** 1.97 to **−** 0.01)**	−**1.46 (**−** 2.39 to **−** 0.53)**	−**2.03 (**−** 2.53 to **−** 1.54)**	−**0.99 (**−** 1.49 to **−** 0.50)**	−0.30 (− 0.72 to 0.12)	−0.17 (− 0.59 to 0.25)
Alcohol-related cancer						
No reported cancer	0.0 (reference)	0.0 (reference)	0.0 (reference)	0.0 (reference)	0.0 (reference)	0.0 (reference)
No/Uncertain	0.55 (− 0.53 to 1.62)	−**1.12 (**−** 2.14 to **−** 0.09)**	−**1.63 (**−** 2.20 to **−** 1.06)**	−**1.08 (**−** 1.66 to **−** 0.51)**	−0.27 (− 0.76 to 0.21)	−0.20 (− 0.69 to 0.29)
Yes	−**6.48 (**−** 8.40 to **−** 4.55)**	−**2.68 (**−** 4.53 to **−** 0.83)**	−**3.49 (**−** 4.23 to **−** 2.75)**	−0.67 (− 1.41 to 0.06)	−0.39 (− 1.05 to 0.27)	−0.06 (− 0.73 to 0.61)

Bolded = *p* <.05

^a^Unadjusted PD

^b^Adjusted PD (aPD) for age category, sex, race/ethnicity, education, insurance, current smoking, marital status, personal healthcare provider, income category

Because some individuals with a cancer history may reduce or stop alcohol consumption while in treatment, we conducted an analysis that excluded those who reported currently being in treatment and found attenuation of prevalence differences for most estimates (Table 3).

**Table 3:** Associations between current drinking and reported cancer history excluding those who reported they were currently being treated, overall and in those 55 years and older

	Current drinking		Binge drinking		Heavy drinking	
	PD (95% CI)^a^	aPD (95% CI)^b^	PD (95% CI)^a^	aPD (95% CI)^b^	PD (95% CI)^a^	aPD (95% CI)^b^
18 years and older (*n* = 461,710)						
Cancer history						
No	0.0 (reference)	0.0 (reference)	0.0 (reference)	0.0 (reference)	0.0 (reference)	0.0 (reference)
Yes	−**4.91 (**− **5.81 to** −**4.00)**	−0.56 (− 1.44 to 0.33)	−**7.57 (**− **8.12 to** −**7.03)**	−**1.11 (**− **1.64 to** −**0.58)**	−**0.67 (**− **1.08 to** −**0.26)**	0.18 (− 0.23 to 0.60)
Alcohol-related cancer						
No reported cancer	0.0 (reference)	0.0 (reference)	0.0 (reference)	0.0 (reference)	0.0 (reference)	0.0 (reference)
No/Uncertain	−**3.61 (**− **4.61 to **−** 2.61)**	−0.29 (− 1.25 to 0.67)	-−**7.08 (**− **7.69 to **− **6.46)**	−**1.33 (**− **1.93 to** −**0.72)**	−**0.56 (**− **1.04 to **−** 0.09)**	0.21 (− 0.27 to 0.68)
Yes	−**10.01 (**− **11.89 to **−** 8.12)**	−1.61 (− 3.41 to 0.19)	−**9.54 (**− **10.50 to **−** 8.58)**	−0.24 (− 1.15 to 0.68)	-−**1.10 (**− **1.81 to** −**0.39)**	0.09 (− 0.63 to 0.81)
55 years and older (*n* = 253,145)						
Cancer history						
No	0.0 (reference)	0.0 (reference)	0.0 (reference)	0.0 (reference)	0.0 (reference)	0.0 (reference)
Yes	−0.51 (− 1.52 to 0.51)	−**0.99 (**− **1.97 to** −**0.01)**	−**1.99 (**− **2.50 to** −**1.48)**	−**0.91 (**− **1.43 to** −**0.40)**	−0.23 (− 0.66 to 0.20)	−**0.10 (**− **0.54 to 0.33)**
Alcohol-related cancer						
No reported cancer	0.0 (reference)	0.0 (reference)	0.0 (reference)	0.0 (reference)	0.0 (reference)	0.0 (reference)
No/Uncertain	0.98 (− 0.13 to 2.10)	−0.72 (− 1.79 to 0.35)	−**1.61 (**− **2.20 to **−** 1.01)**	−**1.03 (**− **1.63 to **−** 0.43)**	−0.22 (− 0.72 to 0.29)	−0.15 (− 0.66 to 0.36)
Yes	−**6.00 (**− **8.05 to **−** 3.95)**	−**1.99 (**− **3.96 to **−** 0.03)**	−**3.39 (**−** 4.17 to **−** 2.60)**	−0.50 (− 1.28 to 0.28)	−0.28 (− 0.99 to 0.43)	0.07 (− 0.65 to 0.78)

Bolded = *p* <.05

^a^Unadjusted PD

^b^Adjusted PD for age category, sex, race/ethnicity, education, insurance, current smoking, marital status, personal healthcare provider, income category

**Fig. 2:**
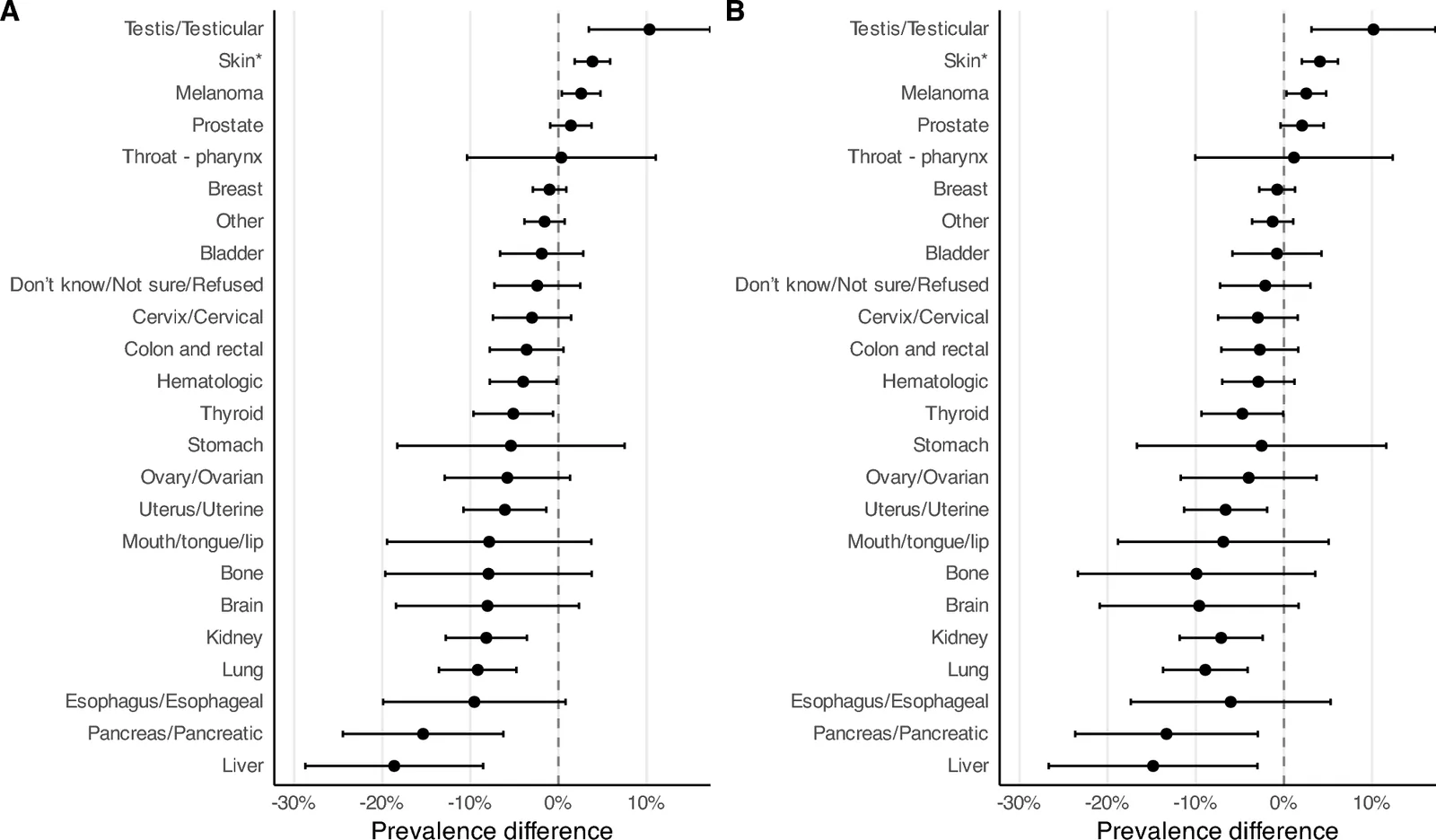
Weighted adjusted prevalence differences between reported cancer type and current drinking, including **A** and excluding those reporting being in current treatment **B**. Models were adjusted for age category, race ethnicity category, insurance category, education category, smoking category, marital category, income category, and personal healthcare provider. Point estimates are statistically significant at *p* <.05 if the 95% CI represented by the line through each point estimate does not cross 0%.*Includes reported non-melanoma skin cancer and types where the participant reported they did not know what kind of skin cancer was diagnosed.

Compared to those without a cancer history, a significantly higher adjusted prevalence of current drinking was observed for individuals with testicular and skin (includes non-melanoma and type of skin cancer unknown) and melanoma skin cancers, while lower prevalences were found for most other cancer types except prostate and throat/pharynx, which were non-significantly higher (Fig. 2A, Supplementary Table 7). When excluding those who reported being in current treatment, 16 of 19 cancer types with aPDs below 1 (Fig. 2B, Supplementary Table 7) moved closer to the null, while 3 of 5 with aPDs above 1 moved further away from the null (Fig. 2B, Supplementary Table 7). When limiting to those 55 and older, the results were generally in the same direction; however, point estimates were generally attenuated (Supplementary Fig. 2, Supplementary Table 8).

## Discussion

We observed a modest, statistically significant decrease over time in the prevalence of current drinking from 2016 to 2024 among those without a cancer history. In contrast, the prevalence of current drinking was generally stable over the study period in those with a cancer history. After covariate adjustment, we found that current and binge drinking had significantly lower prevalences in those with vs. those without a cancer history. The prevalence of current drinking was lower in those with alcohol-related cancers than in both those without alcohol related/uncertain cancers and those with no reported cancer history. In the cancer type analyses, we observed lower prevalences of consuming alcohol for most cancer types, except prostate cancer, melanoma, skin cancer, throat/pharynx cancer, and testicular cancer, although not all were significantly lower. When excluding those in current treatment, most aPDs shifted toward the null of 0 if the aPD was <0 and away from the null if the aPD was >0 when those in current treatment were not excluded, suggesting that many individuals resume alcohol consumption once treatment stops.

Prior trend analyses in those without a cancer history have reported overall increases in alcohol consumption over time. Studies including data collected from 1997 to 2017 indicate that the overall prevalence of drinking has increased [33–38]. For example, current use among adults aged ≥ 60 years rose by an average of 0.7% per year for men and 1.6% per year for women from 1997 to 2014 [34], while another report showed an increase from 65.4% to 72.7% from the period of 2001–2002 to 2012–2013 in adults [35]. Grucza et al. (2018) [39] conducted a meta-analysis of data from six nationally representative U.S. surveys between 2000 and 2016 to assess adult alcohol use and binge drinking trends. Despite prevalence discrepancies between national surveys, the meta-analysis [39] revealed small but significant increases in both past-year alcohol use (average APC: 0.3% per year) and binge drinking (average APC: 0.72% per year). Interestingly, their analysis of BRFSS data from 2001 to 2016, which includes a one-year overlap with our data, provided some evidence for a decline in any alcohol consumption over the last 30 days. Our results using BRFSS data suggest that this trend among those without a cancer history has continued through 2024, but with a very modest decline. Of note, our 2016 weighted prevalence for binge drinking is higher than Grucza’s using the same data. The lower prevalence reported by Grucza et al. [39] may reflect differences in analytic exclusions, imputation, or differential variable measurement.

We found no national studies examining trends in alcohol consumption over time in those with a reported cancer history. Estimates of current drinking from large national surveys reporting cancer survivor-specific estimates [9, 10, 40–42] range between 56.5% [9] and 77.7% [41] and for heavy or exceeding moderate drinking between 5% (2013 to 2017) [42] and 38.04% (1999 to 2016) [10]. For binge drinking, estimates range between 2.8% (2012 to 2017) [40] to 23.8% (2013) [41]. Our estimates were generally lower than other national estimates for current drinking. For example, for current drinking, our estimate for those with a cancer history ranged from 43.6 to 47.2% during 2016 to 2024; however, other comparable studies included earlier years and considered current drinking as having consumed at least one drink in the past year [9, 10, 40, 41] vs. in the last 30 days, which may explain higher prevalences reported in other studies. Sanford et al. [9] compared consumption in individuals with specific cancer types to all other cancer types. Our results were largely similar in direction despite a different reference group of all other cancers, with lower prevalences of current drinking for colorectal, uterine, lung, CNS, and liver, and higher odds for prostate, melanoma, and testicular.

Cancer risks associated with alcohol consumption are becoming increasingly clear, with estimated population attributable fractions varying from 4.4% for female breast cancer to 31.6% for esophageal cancer [43]. However, evidence indicates that most adults are unaware that alcoholic beverage consumption may impact their cancer risk. For example, in a large study using 2020 HINTS data, the authors found that more than 50% of adults were unaware that wine, beer, or liquor increases cancer risk [44]. In a more recent HINTS survey using 2024 data, only 39.4% of respondents reported that alcohol increases cancer risk [15]. A study conducted in Minnesota, including >1700 adults, suggests that awareness varies by cancer type, with the highest and lowest awareness for liver and breast cancer, respectively [45].

Studies examining changes in alcohol use following a cancer diagnosis provide some evidence that a cancer diagnosis changes alcohol consumption behavior [8, 46–48]. For example, in a Canadian study of over 500 survivors, among 299 patients who consumed alcohol at diagnosis, over half (52%) reported less or no consumption one year after diagnosis [46]. In a study of 973 head and neck cancer patients, alcohol consumption declined from 54.3% of patients to 41.2% of patients one year after diagnosis [47]. However, a study of 300 German cancer survivors showed that 70% of patients with a cancer diagnosis continued to drink following diagnosis, measured at six months later [48]. Taken together, these studies suggest that some patients reduce their alcohol use following a cancer diagnosis, although alcohol use is still common. These findings provide one possible explanation for our results that suggest cancer survivors overall and for most cancer types have lower alcohol consumption than those without a cancer history; however, the cross-sectional nature of the BRFSS data prevents concluding that the cancer diagnosis itself led to changes in drinking behavior.

Several national guideline bodies have recommendations for alcohol consumption and screening by healthcare providers, including the American Cancer Society, which states, “It is best not to drink alcohol” [49]. The U.S. Preventive Services Task Force recommends adults be screened for unhealthy alcohol use [50]. The prevalence of alcohol-related discussions between providers and cancer survivors has been reported to be higher than in those without a cancer history (78.4% vs. 74.3%); however, providers may not often discourage unhealthy alcohol use in cancer survivors as one study reported that only 15% of cancer survivors who consumed ≥ 3 drinks per day were advised to reduce their consumption by their provider [17]. There is an opportunity, previously recognized [51], to improve the healthcare provider’s role in increasing patient awareness about the association between alcohol consumption and cancer risk.

Existing evidence indicates that most people remain unaware of alcohol’s link to cancer, which underscores a persistent gap in public awareness [52], and aligns with our results. Given the known cancer-related risks of alcohol consumption as a risk factor for cancer in both those with and without a cancer history, there have been increasing calls for alcoholic beverage warning labels that could increase public awareness [53, 54], including in the United States [12].

Our study has several notable strengths and limitations. A key strength is the use of data from the BRFSS, a large, nationally representative survey that contributes to the external validity of our findings. Additionally, our analytic approach incorporated survey weights and multiple imputation for missing data, which may improve the accuracy and generalizability of the results. However, several limitations should be noted. First, some states were missing survey data for specific years, and not all states included the cancer module, which may limit the generalizability of our prevalence difference analyses. Second, cancer history, cancer type, and alcohol use were self-reported and may be subject to recall, classification, and social desirability bias. Misreporting of a prior cancer diagnosis could lead to misclassification of cancer history. Non-differential misclassification of cancer diagnoses with respect to alcohol consumption would result in an attenuation of prevalence differences, whereas differential misclassification could bias estimates in either direction. Alcohol consumption may also be underreported, potentially leading to underestimation of prevalence. This bias may be particularly relevant to cancer survivors if awareness of alcohol-related health risks increases the tendency to under-report drinking after diagnosis. Similarly, changes in reporting or under-reporting over time could influence observed temporal trends. Third, the sample size for some cancer types was small, reducing the precision of our estimates. Fourth, we assumed that missing data were missing at random; while imputation can mitigate bias under this assumption, it cannot address data that are not missing at random. Fifth, several assumptions were made in the post-imputation logic, which may not fully reflect the underlying data structure. Our results should be interpreted with these limitations in mind.

## Conclusions and future directions

In conclusion, recent alcohol consumption showed a very modest decline among individuals without a cancer history but remained stable among those with a reported cancer history. Although individuals with a cancer history generally had a lower prevalence of current drinking, attenuation of these differences after excluding those in current treatment suggests that these patterns may reflect, in part, temporary changes during treatment. These findings add to the evidence on alcohol use patterns among U.S. adults with and without a cancer history and may be relevant to future examination of trends and differences in alcohol consumption in these groups.

## Supplementary Information

Below is the link to the electronic supplementary material.

Supplementary file1 (DOCX 162 KB)

Supplementary file2 (DOCX 178 KB)

## Data Availability

The datasets analysed during the current study are available on the BRFSS website: https://www.cdc.gov/brfss/annual_data/annual_data.htm and from the corresponding author on request. The code used to analyze these data can be found at: https://github.com/kijohnson/Alcohol-Cancer-BRFSS-paper.
