# Supplementary material for: Prevalence and trends in recent alcohol consumption by cancer history and cancer type in a nationally representative sample of U.S. adults
Source: Cancer Causes Control. 2026 Sep 24;37(10):167. doi: 10.1007/s10552-026-02248-8 (PMC13612666; doi:10.1007/s10552-026-02248-8)
Supplement: Supplementary file 1 [file 10552_2026_2248_MOESM1_ESM.docx]

**Supplementary Materials**

**Supplementary Table 1.** Respondent states/territories included from 2022, 2023, and 2024 BRFSS surveys^a^ that included the “Cancer Survivorship: Type of Cancer” optional module in prevalence difference analyses.

| **Year** | **File** | **State** |
| --- | --- | --- |
| 2022 | LLCP2022 | Arkansas, Colorado, Connecticut, Delaware, Hawaii, Idaho, Illinois, Indiana, Michigan, Missouri, Nebraska, Nevada, New Jersey, New Mexico, North Carolina, Oregon, Puerto Rico, Rhode Island, South Dakota, Utah, Vermont, Virginia, Wisconsin |
|  | LLCP22V1 | Iowa, Massachusetts, Ohio, Oklahoma |
|  | LLCP22V2 | Arizona, Kansas |
|  | LLCP22V3 | Maryland |
| 2023 | LLCP2023 | Connecticut, Georgia, Illinois, Indiana, Michigan, Minnesota, Mississippi, New Hampshire, New Jersey, Tennessee, Virgin Islands, Virginia, West Virginia, Wisconsin |
|  | LLCP23V1 | New York, Oklahoma |
|  | LLCP23V2 | Kansas, Maryland |
| 2024 | LLCP2024 | Connecticut, Delaware, Georgia, Indiana, Massachusetts, Michigan, Missouri, New Jersey, North Carolina, South Dakota, Utah, Virgin Islands, Virginia |
|  | LLCP24V1 | Arizona, Kansas |
|  | LLCP24V2 | Nebraska, New York |

**^a^**Survey data can be found at: https://www.cdc.gov/brfss/data_documentation/index.htm

**Supplementary Table 2.** Description of BRFSS variables used to harmonize and derive variables for analyses.

| **BRFSS Variable name** | **Year(s)** | **Question/Description** | **Coding** | **Derived variable** |
| --- | --- | --- | --- | --- |
| ALCDAY4 | 2022-2024 | During the past 30 days, how many days per week or  per month did you have at least one drink of any alcoholic beverage?  (A 40ounce beer would count as 3 drinks, or a cocktail  drink with 2 shots would count as 2 drinks.) | <300 = Yes (at least one drink in the last 30 days; the values can range from 101 to 107 for days per week specified in the last 30 days or from 201 to 230 for days per month in the last 30 days specified)  888 = No (no drinks in last 30 days)  999 (Refused), 777, or blank = missing | Current drinking |
| ALCDAY5 | 2016-2021 | During the past 30 days, how many days per week or per month did you have at least one drink of any alcoholic beverage such as beer, wine, a malt beverage or liquor? | <300 = Yes (at least one drink in the last 30 days; the values can range from 101 to 107 for days per week specified in the last 30 days or from 201 to 230 for days per month in the last 30 days specified)  888 = No (no drinks in last 30 days)  999 (Refused) or blank = missing | Current drinking |
| _RFBING6 | 2022-2024 | Binge drinkers (males having five or more drinks on one occasion, females having four or more drinks on one  occasion) | 1 = No  2 = Yes  9 (Don’t know/refused/missing) = missing  This is a derived variable from ALCDAY4 with ALCDAY4 <231 and DRNK3GE5 = 88 or ALCDAY4 = 888 indicating not a binge drinker and ALCDAY4 <231 and 1 ≤ DRNK3GE5 ≤ 76 indicating a binge drinker. | Binge drinking |
| _RFBING5 | 2016-2021 | Binge drinkers (males having five or more drinks on one occasion, females having four or more drinks on one occasion) | 1 = No  2 = Yes  9 (Don’t know/refused/missing) = missing  This is a derived variable from ALCDAY5 with ALCDAY5 <300 and DRNK3GE5 = 1 or ALCDAY5 = 888 indicating not a binge drinker and ALCDAY5 <300 and DRNK3GE5 = 1 indicating a binge drinker. | Binge drinking |
| _RFDRHV5 | 2016-2017 | Heavy drinkers (adult men having more than 14drinks  per week and adult women having more than 7 drinks per week) | <300 = Yes (at least one drink in the last 30 days; the values can range from 101 to 107 for days per week specified in the last 30 days or from 201 to 230 for days per month in the last 30 days specified)  888 = No (no drinks in last 30 days)  999 (Refused), 777, or blank = missing | Heavy drinking |
| _RFDRHV6 | 2018 | Heavy drinkers (adult men having more than 14drinks  per week and adult women having more than 7 drinks per week) | 1 = No  2 = Yes  9 (Don’t know/refused/missing) = missing | Heavy drinking |
| _RFDRHV7 | 2019-2021 | Heavy drinkers (adult men having more than 14drinks  per week and adult women having more than 7 drinks per week) | 1 = No  2 = Yes  9 (Don’t know/refused/missing) = missing | Heavy drinking |
| _RFDRHV9 | 2024 | Heavy drinkers (adult men having more than 14 drinks per week and adult women having more than 7 drinks per week) | 1 = No  2 = Yes  9 = (Don’t know/refused/missing) = missing | Heavy drinking |
| _RFDRHV8 | 2022-2023 | Heavy drinkers (adult men having more than 14 drinks per week and adult women having more than 7  drinks per week) | 1 = No  2 = Yes  9 (Don’t know/refused/missing) = missing | Heavy drinking |
| CHCOCNC1 | 2022-2024 | (Ever told) (you had) melanoma or any other types of  cancer? | 1 = Yes  2 = No  7, 9, or blank (Don’t know/Not sure/Refused/Blank) = Missing | Reported cancer history |
| CHCOCNCR | 2016-2021 | (Ever told) you had any other types of cancer? | 1 = Yes  2 = No  7, 9, or blank (Don’t know/Not sure/Refused/Blank) = Missing | Reported cancer history (yes/no) |
| CSRVTRT3 | 2022-2024 | Are you currently receiving treatment for cancer? | 1 = Yes  2, 3, 4, 5 = No  7, 9, or blank (Don’t know/Not sure/Refused/Blank) = Missing | Current treatment |
| CNCRTYP2 | 2022-2024 | What kind of cancer is it? | Codes 0 to 32 with 0 being no cancer  77, 99, or blank (Don’t know/Not sure/Refused/Blank) = Missing | Cancer type |
| _AGE_G | 2016-2024 | Six-level imputed age category | 1 = 18 to 24  2 = 25 to 34  3 = 35 to 44  4 = 45 to 54  5 = 55 to 64  6 = 65 and older | Age category |
| SEXVAR | 2019-2024 | Sex of Respondent | 1 = Male  2 = Female | Sex |
| SEX1 | 2018 | What is your sex? or What was your sex at birth? Was it… | 1 = Male  2 = Female | Sex |
| SEX | 2016-2017 | Indicate sex of respondent | 1 = Male  2 = Female | Sex |
| _RACEPRV | 2020, 2021, 2023-2024 | Race/ethnicity categories | 1 = Non-Hispanic White  2 = Non-Hispanic Black  3 = Non-Hispanic American Indian/Alaskan Native  4 = Non-Hispanic Asian  5 = Non-Hispanic Asian Pacific Islander  6,7 = Non-Hispanic Other/Multi  8 = Hispanic  Blank = Missing | Race/ethnicity |
| _RACEPR1 | 2022 | Race/ethnicity categories | 1 = Non-Hispanic White  2 = Non-Hispanic Black  3 = Non-Hispanic American Indian/Alaskan Native  4 = Non-Hispanic Asian  5 = Non-Hispanic Asian Pacific Islander  6 = Non-Hispanic Other/Multi  7 = Hispanic | Race/ethnicity |
| _RACE | 2016-2019 | Race/ethnicity categories | 1 = Non-Hispanic White  2 = Non-Hispanic Black  3 = Non-Hispanic American Indian/Alaskan Native  4 = Non-Hispanic Asian  5 = Non-Hispanic Asian Pacific Islander  6, 7 = Non-Hispanic Other/Multi  8 = Hispanic  9 or blank (Don’t know/Not sure/Refused/Blank) = Missing | Race/ethnicity |
| _RFSMOK3 | 2016-2024 | Adults who are current smokers | 1 = No  2 = Yes  9 or blank (Don’t know/Not sure/Refused/Blank) = Missing | Current smoking |
| EDUCA | 2016-2024 | What is the highest grade or year of school you completed? | 1, 2, 3 = < High school  4 = High school or GED  5 = Some college  6 = College or more  9 or blank (Refused/Blank) = Missing | Education |
| _HLTHPL2 | 2024 | Adults who had some form of health insurance | 1 = Insurance  2 = No insurance  9 or blank (Don’t know/Not sure/Refused/Blank) = Missing | Insurance |
| _HLTHPL1 | 2023 | Adults who had some form of health insurance | 1 = Insurance  2 = No insurance  9 or blank (Don’t know/Not sure/Refused/Blank) = Missing | Insurance |
| _HLTHPLN | 2021-2022 | Adults who had some form of health insurance | 1 = Insurance  2 = No insurance  9 or blank (Don’t know/Not sure/Refused/Blank) = Missing | Insurance |
| HLTHPLN1 | 2016-2020 | Do you have any kind of health care coverage, including health insurance, prepaid plans such as HMOs, or government plans such as Medicare, or Indian Health Service? | 1 = Yes  2 = No  9 or blank (Don’t know/Not sure/Refused/Blank) = Missing | Insurance |
| MARITAL | 2016-2024 | Are you: (marital status) | 1 = Married  2, 3, 4, 5, 6 = Unmarried  9 or blank = Missing | Marital status |
| _INCOMG1 | 2021-2024 | Income categories | 1, 2, 3, 4 = <50K  5, 6, 7 = 50K+  9 or blank (Don’t know/Not sure/Refused/Blank) = Missing | Income |
| _INCOMG | 2016-2020 | Income categories | 1, 2, 3, 4 = <50K  5 = 50K+  9 or blank (Don’t know/Not sure/Refused/Blank) = Missing | Income |
| PERSDOC3 | 2021-2024 | Do you have one person or a group of doctors that you  think of as your personal health care provider? | 1, 2 = Yes  3 = No  7, 9, or blank (Don’t know/Not sure/Refused/Blank) = Missing | Personal healthcare provider |
| PERSDOC2 | 2016-2020 | Do you have one person you think of as your personal  doctor or health care provider? | 1, 2 = Yes  3 = No  7, 9, or blank (Don’t know/Not sure/Refused/Blank) = Missing | Personal healthcare provider |

**Supplementary Table 3.** Missing data for variables included in models in 2016–2024 and 2022–2024.

|  | **Trend analyses**  **(2016 to 2024)** | | **Prevalence difference analyses**  **(2022 to 2024)** | |
| --- | --- | --- | --- | --- |
| **Variable** | **No. missing** | **Percent missing** | **No. missing** | **Percent**  **missing** |
| Income | 756848 | 19.1 | 97220 | 20.9 |
| Current drinking | 272928 | 6.9 | 44361 | 9.5 |
| Binge drinking | 311283 | 7.8 | 48608 | 10.4 |
| Heavy drinking | 309115 | 7.8 | 47530 | 10.2 |
| Current smoking | 210912 | 5.3 | 33556 | 7.2 |
| Insurance | 82281 | 2.1 | 19240 | 4.1 |
| Race/ethnicity | 34941 | 0.9 | 16 | 0 |
| Marital status | 35295 | 0.9 | 4522 | 1 |
| Personal healthcare provider | 27444 | 0.7 | 4412 | 0.9 |
| Education | 18373 | 0.5 | 2421 | 0.5 |
| Reported cancer history | 14447 | 0.4 | 2495 | 0.5 |
| Current treatment | NA | NA | 433598 | 93.1 |
| Cancer type | NA | NA | 9265 | 2 |
| Sex | 1463 | 0 | 0 | 0 |
| Age category | 0 | 0 | 0 | 0 |

**Supplementary Table 4.** Distribution of cancer types in BRFSS participants in 2022 and 2024 based on imputed data.

|  | **Including those who reported being in current treatment** | | **Excluding those who reported being in current treatment** | |
| --- | --- | --- | --- | --- |
| **Cancer Type** | **Mean N (Range)** | **Mean % of Total Cancers (Range)** | **Mean N (Range)** | **Mean % of Total Cancers (Range)** |
| None | 412316 (412268-412343) | NA | 411657 (411608-411678) | NA |
| Bladder | 1198 (1195-1201) | 2.2% (2.2-2.2) | 1066 (1061-1075) | 2.1% (2.1-2.2) |
| Bone | 222 (215-237) | 0.4% (0.4-0.4) | 172 (164-186) | 0.3% (0.3-0.4) |
| Brain | 215 (212-220) | 0.4% (0.4-0.4) | 182 (178-189) | 0.4% (0.4-0.4) |
| Breast | 8467 (8430-8511) | 15.8% (15.7-15.9) | 7542 (7487-7614) | 15.2% (15.1-15.3) |
| Cervix/Cervical | 1378 (1374-1381) | 2.6% (2.6-2.6) | 1343 (1339-1347) | 2.7% (2.7-2.7) |
| Colon and rectal | 2184 (2174-2201) | 4.1% (4.1-4.1) | 2037 (2025-2055) | 4.1% (4.1-4.1) |
| Don’t know/Not sure/Refused | 1333 (1311-1353) | 2.5% (2.4-2.5) | 1221 (1202-1245) | 2.5% (2.4-2.5) |
| Esophagus/Esophageal | 195 (191-202) | 0.4% (0.4-0.4) | 166 (162-172) | 0.3% (0.3-0.3) |
| Hematologic | 2367 (2359-2379) | 4.4% (4.4-4.4) | 2060 (2053-2069) | 4.1% (4.1-4.2) |
| Kidney | 1025 (1009-1037) | 1.9% (1.9-1.9) | 959 (944-971) | 1.9% (1.9-2) |
| Liver | 220 (216-226) | 0.4% (0.4-0.4) | 173 (168-178) | 0.3% (0.3-0.4) |
| Lung | 1462 (1429-1509) | 2.7% (2.7-2.8) | 1257 (1222-1309) | 2.5% (2.5-2.6) |
| Melanoma | 6883 (6865-6909) | 12.9% (12.8-12.9) | 6608 (6589-6639) | 13.3% (13.3-13.4) |
| Mouth/tongue/lip | 162 (156-179) | 0.3% (0.3-0.3) | 154 (148-170) | 0.3% (0.3-0.3) |
| Other | 9342 (9297-9410) | 17.4% (17.4-17.6) | 8952 (8890-8994) | 18% (18-18.1) |
| Ovary/Ovarian | 626 (620-632) | 1.2% (1.2-1.2) | 576 (568-584) | 1.2% (1.1-1.2) |
| Pancreas/Pancreatic | 242 (241-244) | 0.5% (0.4-0.5) | 200 (197-202) | 0.4% (0.4-0.4) |
| Prostate | 5290 (5260-5337) | 9.9% (9.8-10) | 4748 (4715-4791) | 9.6% (9.5-9.6) |
| Skin^a^ | 7685 (7643-7726) | 14.4% (14.3-14.4) | 7322 (7290-7359) | 14.7% (14.7-14.8) |
| Stomach | 172 (169-178) | 0.3% (0.3-0.3) | 148 (144-154) | 0.3% (0.3-0.3) |
| Testis/Testicular | 272 (271-273) | 0.5% (0.5-0.5) | 263 (261-265) | 0.5% (0.5-0.5) |
| Throat - pharynx | 268 (263-274) | 0.5% (0.5-0.5) | 255 (249-260) | 0.5% (0.5-0.5) |
| Thyroid | 1210 (1200-1219) | 2.3% (2.2-2.3) | 1147 (1138-1152) | 2.3% (2.3-2.3) |
| Uterus/Uterine | 1136 (1128-1147) | 2.1% (2.1-2.1) | 1097 (1084-1115) | 2.2% (2.2-2.2) |

^a^Includes reported non-melanoma skin cancer and types where the participant reported they did not know what kind of skin cancer was diagnosed.

**Supplementary Table 5.** Weighted and unweighted prevalences of current drinking, binge drinking, and heavy drinking.

|  |  | **Current drinking** | | **Binge drinking** | | **Heavy drinking** | |
| --- | --- | --- | --- | --- | --- | --- | --- |
| **Reported**  **cancer?** | **Year** | **Unweighted**  **Prevalence (%)**  **(95% CI)** | **Weighted**  **Prevalence (%)**  **(95% CI)** | **Unweighted**  **Prevalence (%)**  **(95% CI)** | **Weighted**  **Prevalence (%)**  **(95% CI)** | **Unweighted**  **Prevalence (%)**  **(95% CI)** | **Weighted**  **Prevalence (%)**  **(95% CI)** |
| No | 2016 | 52.3 (52–52.6) | 53.9 (53.6–54.3) | 13.9 (13.7–14) | 17.7 (17.4–18) | 6.1 (6–6.2) | 6.5 (6.4–6.7) |
| Yes | 2016 | 44.5 (44–45) | 45.8 (44.7–46.8) | 6.6 (6.3–6.8) | 8.4 (7.8–9.1) | 4.9 (4.7–5.1) | 5.2 (4.7–5.7) |
| No | 2017 | 52.9 (52.6–53.2) | 54.4 (54–54.8) | 14.3 (14.2–14.5) | 17.9 (17.6–18.2) | 6 (5.9–6.1) | 6.4 (6.2–6.6) |
| Yes | 2017 | 45.9 (45.4–46.3) | 46.9 (45.8–48) | 6.9 (6.6–7.1) | 9.1 (8.4–9.8) | 4.8 (4.6–5) | 5.4 (4.8–5.9) |
| No | 2018 | 52.4 (52.2–52.5) | 53.2 (52.9–53.6) | 14.4 (14.3–14.5) | 17.2 (17–17.5) | 6.3 (6.2–6.4) | 6.5 (6.3–6.7) |
| Yes | 2018 | 44.8 (44.3–45.3) | 45 (43.8–46.1) | 6.7 (6.5–7) | 8 (7.4–8.6) | 4.8 (4.6–5) | 5 (4.6–5.5) |
| No | 2019 | 51.4 (51.2–51.6) | 53.2 (52.9–53.5) | 14.3 (14.2–14.4) | 17.6 (17.3–17.9) | 6.2 (6.1–6.3) | 6.6 (6.4–6.7) |
| Yes | 2019 | 43.9 (43.4–44.4) | 45.2 (44.1–46.3) | 7 (6.7–7.2) | 9 (8.3–9.8) | 4.8 (4.6–5) | 5.6 (5–6.1) |
| No | 2020 | 51.9 (51.6–52.2) | 52.4 (51.9–52.8) | 14.3 (14.2–14.4) | 16.4 (16.1–16.6) | 6.7 (6.6–6.8) | 6.8 (6.5–7) |
| Yes | 2020 | 44.2 (43.6–44.8) | 44.7 (43.4–45.9) | 6.6 (6.3–6.9) | 7.9 (7.3–8.6) | 5.1 (4.8–5.4) | 5.5 (4.9–6.1) |
| No | 2021 | 52.6 (52.4–52.9) | 52.8 (52.4–53.2) | 14.3 (14.1–14.4) | 16.4 (16.1–16.7) | 6.1 (6–6.2) | 6.2 (6–6.4) |
| Yes | 2021 | 44.7 (43.9–45.5) | 43.9 (42.5–45.2) | 6.8 (6.5–7) | 8 (7.3–8.8) | 4.7 (4.4–4.9) | 4.8 (4.3–5.2) |
| No | 2022 | 53.7 (53.6–53.9) | 53.1 (52.7–53.5) | 15.5 (15.4–15.7) | 17.5 (17.2–17.8) | 6.8 (6.7–6.9) | 6.7 (6.5–6.9) |
| Yes | 2022 | 48.5 (47.4–49.6) | 46.8 (45.7–48) | 7.9 (7.7–8.1) | 9.3 (8.6–9.9) | 5.5 (5.3–5.7) | 5.8 (5.3–6.3) |
| No | 2023 | 53.3 (53.1–53.6) | 52.2 (51.8–52.6) | 14.3 (14.2–14.4) | 16.1 (15.8–16.4) | 6.1 (6–6.2) | 5.9 (5.7–6.1) |
| Yes | 2023 | 49.1 (48.6–49.5) | 47.4 (46.4–48.5) | 7 (6.8–7.3) | 8.4 (7.6–9.1) | 5 (4.8–5.2) | 5 (4.6–5.4) |
| No | 2024 | 51.4 (51.1–51.7) | 51.5 (51.1–51.8) | 14.4 (14.2–14.5) | 16.2 (15.9–16.5) | 6 (5.8–6.1) | 5.8 (5.6–6) |
| Yes | 2024 | 46.6 (46–47.3) | 45.8 (44.8–46.9) | 7.3 (7–7.5) | 8.9 (8.3–9.5) | 5 (4.8–5.2) | 5.3 (4.8–5.7) |

**Supplementary Table 6.** Linear regression model results for trend analyses.

| **AAPC (95% CI)** | **p-value** | **Model** |
| --- | --- | --- |
| -0.28 (-0.42 to -0.14) | 0.002 | No reported cancer/current drinking |
| 0.06 (-0.31 to 0.43) | 0.698 | Reported cancer/current drinking |
| -0.20 (-0.35 to -0.05) | 0.017 | No reported cancer/binge drinking |
| 0.025 (-0.15 to 0.20) | 0.741 | Reported cancer/binge drinking |
| -0.072 (-0.16 to 0.02) | 0.111 | No reported cancer/heavy drinking |
| 0.000 (-0.11 to 0.11) | 1.000 | Reported cancer/heavy drinking |

**Supplementary Table 7.** Weighted adjusted prevalence difference estimates for current drinking by cancer type (reference = No reported cancer history) including and excluding those with a cancer history who reported being in current treatment.

|  | **Including those in current treatment** | **Excluding those in current treatment^a^** | **Closer to the null?** |
| --- | --- | --- | --- |
| **Cancer site** | **PD (%) (95% CI)^b^** | **PD (%) (95% CI)^b^** |  |
| Bladder | -1.9 (-2.99 to -0.8) | -0.79 (-1.89 to 0.31) | Yes |
| Bone | -7.94 (-13.4 to -2.48) | -9.92 (-16.62 to -3.21) | No |
| Brain | -8.05 (-9.03 to -7.07) | -9.61 (-10.59 to -8.62) | No |
| Breast | -1.01 (-1.48 to -0.55) | -0.76 (-1.25 to -0.27) | Yes |
| Cervix/Cervical | -3 (-4.38 to -1.62) | -2.95 (-4.37 to -1.54) | Yes |
| Colon and rectal | -3.61 (-4.96 to -2.26) | -2.74 (-4.13 to -1.34) | Yes |
| Don’t know/Not sure/Refused | -2.4 (-3.91 to -0.9) | -2.11 (-3.78 to -0.45) | Yes |
| Esophagus/Esophageal | -9.54 (-10.94 to -8.15) | -6.03 (-7.6 to -4.47) | Yes |
| Hematologic | -4 (-4.53 to -3.48) | -2.9 (-3.43 to -2.36) | Yes |
| Kidney | -8.19 (-9.49 to -6.89) | -7.11 (-8.4 to -5.83) | Yes |
| Liver | -18.64 (-21.61 to -15.67) | -14.84 (-18.55 to -11.13) | Yes |
| Lung | -9.16 (-9.93 to -8.39) | -8.92 (-10 to -7.83) | Yes |
| Melanoma | 2.58 (2.26 to 2.91) | 2.54 (2.16 to 2.92) | Yes |
| Mouth/tongue/lip | -7.86 (-10.8 to -4.92) | -6.88 (-10.03 to -3.72) | Yes |
| Other | -1.57 (-3.06 to -0.09) | -1.28 (-2.81 to 0.25) | Yes |
| Ovary/Ovarian | -5.8 (-7.37 to -4.23) | -3.99 (-5.8 to -2.19) | Yes |
| Pancreas/Pancreatic | -15.36 (-16.86 to -13.85) | -13.32 (-15.69 to -10.95) | Yes |
| Prostate | 1.41 (1.08 to 1.75) | 2.06 (1.77 to 2.36) | No |
| Skin^c^ | 3.86 (3.39 to 4.32) | 4.09 (3.6 to 4.57) | No |
| Stomach | -5.39 (-9.6 to -1.18) | -2.53 (-7.42 to 2.36) | Yes |
| Testis/Testicular | 10.33 (8.37 to 12.29) | 10.17 (8.13 to 12.2) | Yes |
| Throat - pharynx | 0.33 (-1.24 to 1.89) | 1.14 (-0.64 to 2.92) | No |
| Thyroid | -5.12 (-6.64 to -3.61) | -4.71 (-6.27 to -3.16) | Yes |
| Uterus/Uterine | -6.08 (-6.91 to -5.24) | -6.61 (-7.32 to -5.9) | No |

^a^n = 4,160 excluded who reported being in current treatment in the parent dataframe for the imputation (no imputed values included)

^b^Adjusted for Models were adjusted for age category, race ethnicity category, insurance category, education category, smoking category, marital category, income category, and personal healthcare provider.

^c^Includes reported non-melanoma skin cancer and types where the participant reported they did not know what kind of skin cancer was diagnosed.

**Supplementary Table 8.** Weighted adjusted prevalence difference estimates for current drinking by cancer type (reference = No reported cancer history) including and excluding those with a cancer history who reported being in current treatment in those 55 and older.

|  | **Including those in current treatment** | **Excluding those in current treatment^a^** | **Closer to the null?** |
| --- | --- | --- | --- |
| **Cancer site** | **PD (%) (95% CI)^b^** | **PD (%) (95% CI)^b^** |  |
| Bladder | -2.74 (-7.64 to 2.15) | -1.47 (-6.75 to 3.81) | Yes |
| Bone | -2.99 (-16.4 to 10.41) | -5.09 (-21.21 to 11.02) | No |
| Brain | 1.39 (-12.15 to 14.94) | 1.76 (-13.12 to 16.63) | No |
| Breast | -1.81 (-3.88 to 0.26) | -1.45 (-3.64 to 0.74) | Yes |
| Cervix/Cervical | -6.64 (-12.11 to -1.18) | -6.85 (-12.4 to -1.31) | No |
| Colon and rectal | -4.25 (-8.72 to 0.22) | -3.35 (-8.03 to 1.32) | Yes |
| Don’t know/Not sure/Refused | -1.6 (-6.98 to 3.77) | -1.34 (-7.04 to 4.35) | Yes |
| Esophagus/Esophageal | -8.2 (-19.38 to 2.98) | -3.78 (-15.96 to 8.41) | Yes |
| Hematologic | -5.07 (-9.42 to -0.72) | -3.71 (-8.47 to 1.06) | Yes |
| Kidney | -8.8 (-13.66 to -3.93) | -8.31 (-13.32 to -3.29) | Yes |
| Liver | -25.71 (-35.2 to -16.21) | -23.83 (-35.26 to -12.4) | Yes |
| Lung | -8.39 (-12.48 to -4.31) | -7.74 (-12.05 to -3.43) | Yes |
| Melanoma | 2.35 (-0.01 to 4.71) | 2.26 (-0.18 to 4.69) | Yes |
| Mouth/tongue/lip | -3.83 (-15.59 to 7.92) | -2.75 (-14.87 to 9.37) | Yes |
| Other | -2.41 (-5.03 to 0.21) | -2.14 (-4.83 to 0.55) | Yes |
| Ovary/Ovarian | -8.83 (-16.76 to -0.9) | -7.65 (-16.42 to 1.11) | Yes |
| Pancreas/Pancreatic | -13.6 (-23.52 to -3.68) | -11.81 (-23.13 to -0.5) | Yes |
| Prostate | 1.44 (-0.94 to 3.81) | 2.03 (-0.43 to 4.5) | No |
| Skin^c^ | 2.46 (0.38 to 4.54) | 2.66 (0.52 to 4.79) | No |
| Stomach | -0.9 (-17.16 to 15.37) | 0.27 (-17.27 to 17.81) | Yes |
| Testis/Testicular | 2.97 (-7.87 to 13.81) | 2.28 (-8.66 to 13.21) | Yes |
| Throat - pharynx | 0.66 (-11.29 to 12.61) | 2.37 (-10.19 to 14.92) | No |
| Thyroid | -4.72 (-10.12 to 0.68) | -4.22 (-9.76 to 1.32) | No |
| Uterus/Uterine | -5.13 (-9.97 to -0.28) | -5.19 (-10.1 to -0.28) | Yes |

^a^n = 3,668 excluded who reported being in current treatment in the parent data frame for the imputation (no imputed values included)

^b^Adjusted for Models were adjusted for age category, race ethnicity category, insurance category, education category, smoking category, marital category, income category, and personal healthcare provider.

^c^Includes reported non-melanoma skin cancer and types where the participant reported they did not know what kind of skin cancer was diagnosed.

**Supplementary Figure 1** Unweighted prevalences by reported cancer history of (**A**) current drinking, (**B**) heavy drinking, and (**C**) binge drinking.

**Supplementary Figure 2** Weighted-adjusted prevalence differences between reported cancer type and current drinking, including (**A**) and excluding (**B**) those reporting being in current treatment in those 55 and older. Models were adjusted for age category, race/ethnicity category, insurance category, education category, smoking category, marital category, income category, and personal healthcare provider. Point estimates are statistically significant at p < .05 if the 95% CI represented by the line through each point estimate does not cross 0%.*Includes reported non-melanoma skin cancer and types where the participant reported they did not know what kind of skin cancer was diagnosed.
