# Supplementary material for: Prevalence and trends in recent alcohol consumption by cancer history and cancer type in a nationally representative sample of U.S. adults
Source: Cancer Causes Control. 2026 Sep 24;37(10):167. doi: 10.1007/s10552-026-02248-8 (PMC13612666; doi:10.1007/s10552-026-02248-8)
Supplement: Supplementary file 2 [file 10552_2026_2248_MOESM2_ESM.docx]

**Supplementary Materials**

**METHODS**

*Imputation and post-imputation logic.* Missing data were addressed using multiple imputation by chained equations, conducted separately within survey year to preserve year-specific distributions. The imputation model for the trend analysis using 2016 to 2024 data included the following variables: reported cancer history, current drinking, age category, race/ethnicity, sex, binge drinking, heavy drinking, insurance status, education, current smoking, marital status, income, year, and personal healthcare provider. A total of 3 imputation dataframes were created. For the prevalence difference analyses using 2022 to 2024 data, we conducted multiple imputation, generating 5 datasets. The imputation model included the same variables as the trend analysis, plus current treatment, but excluded cancer history and cancer type as predictors due to model error issues. Completed datasets were then combined across years and analyzed using survey-weighted methods, with estimates pooled across imputations using Rubin’s rules.

Following multiple imputation, post-imputation logic was applied to address implausible combinations of values or values that would not be found because of survey skip patterns. For both datasets, because binge and heavy drinking are defined over the past 30 days, respondents who reported no alcohol use in the past 30 days necessarily could not meet the criteria for asking about binge or heavy drinking. Therefore, any imputed binge/heavy drinking among past-30-day non-drinkers was recoded to 0 to align with the intended construct and BRFSS skip patterns.

For the 2022 to 2024 imputed datasets, we classified individuals who did not have a reported cancer history as having a cancer type of none, and those with a reported sex of Male who had a cancer type as cervix/cervical, uterus/uterine, or ovary/ovarian as “Other”. Similarly, for females, reclassified those with a reported cancer type as prostate or testes/testicular as “Other”. We also classified those with a reported cancer history and a cancer type value of none as having cancer type “Other”. Alcohol-related cancers were defined following imputation. We excluded individuals who reported being in current treatment in the original data (not who were imputed to be in current treatment) in analyses that estimated prevalence differences excluding those in current treatment

**TABLES AND FIGURES**

**Supplementary Table 1.** Respondent states/territories included from 2022, 2023, and 2024 BRFSS surveys^a^ that included the “Cancer Survivorship: Type of Cancer” optional module in prevalence difference analyses.

| **Year** | **File** | **State** |
| --- | --- | --- |
| 2022 | LLCP2022 | Arkansas, Colorado, Connecticut, Delaware, Hawaii, Idaho, Illinois, Indiana, Michigan, Missouri, Nebraska, Nevada, New Jersey, New Mexico, North Carolina, Oregon, Puerto Rico, Rhode Island, South Dakota, Utah, Vermont, Virginia, Wisconsin |
|  | LLCP22V1 | Iowa, Massachusetts, Ohio, Oklahoma |
|  | LLCP22V2 | Arizona, Kansas |
|  | LLCP22V3 | Maryland |
| 2023 | LLCP2023 | Connecticut, Georgia, Illinois, Indiana, Michigan, Minnesota, Mississippi, New Hampshire, New Jersey, Tennessee, Virgin Islands, Virginia, West Virginia, Wisconsin |
|  | LLCP23V1 | New York, Oklahoma |
|  | LLCP23V2 | Kansas, Maryland |
| 2024 | LLCP2024 | Connecticut, Delaware, Georgia, Indiana, Massachusetts, Michigan, Missouri, New Jersey, North Carolina, South Dakota, Utah, Virgin Islands, Virginia |
|  | LLCP24V1 | Arizona, Kansas |
|  | LLCP24V2 | Nebraska, New York |

**^a^**Survey data can be found at: https://www.cdc.gov/brfss/data_documentation/index.htm

**Supplementary Table 2.** Description of BRFSS variables used to harmonize and derive variables for analyses.

| **BRFSS Variable name** | **Year(s)** | **Question/Description** | **Coding** | **Derived variable** |
| --- | --- | --- | --- | --- |
| ALCDAY4 | 2022-2024 | During the past 30 days, how many days per week or  per month did you have at least one drink of any alcoholic beverage?  (A 40ounce beer would count as 3 drinks, or a cocktail  drink with 2 shots would count as 2 drinks.) | <300 = Yes  888 = No  999 (Refused) or blank = missing | Current drinking |
| ALCDAY5 | 2016-2021 | During the past 30 days, how many days per week or per month did you have at least one drink of any alcoholic beverage such as beer, wine, a malt beverage or liquor? | <300 = Yes  888 = No  999 (Refused) or blank = missing | Current drinking |
| _RFBING6 | 2022-2024 | Binge drinkers (males having five or more drinks on one occasion, females having four or more drinks on one  occasion) | 1 = No  2 = Yes  9 (Don’t know/refused/missing) = missing | Binge drinking |
| _RFBING5 | 2016-2021 | Binge drinkers (males having five or more drinks on one occasion, females having four or more drinks on one occasion) | 1 = No  2 = Yes  9 (Don’t know/refused/missing) = missing | Binge drinking |
| _RFDRHV5 | 2016-2017 | Heavy drinkers (adult men having more than 14drinks  per week and adult women having more than 7 drinks per week) | 1 = No  2 = Yes  9 (Don’t know/refused/missing) = missing | Heavy drinking |
| _RFDRHV6 | 2018 | Heavy drinkers (adult men having more than 14drinks  per week and adult women having more than 7 drinks per week) | 1 = No  2 = Yes  9 (Don’t know/refused/missing) = missing | Heavy drinking |
| _RFDRHV7 | 2019-2021 | Heavy drinkers (adult men having more than 14drinks  per week and adult women having more than 7 drinks per week) | 1 = No  2 = Yes  9 (Don’t know/refused/missing) = missing | Heavy drinking |
| _RFDRHV9 | 2024 | Heavy drinkers (adult men having more than 14 drinks per week and adult women having more than 7 drinks per week) | 1 = No  2 = Yes  9 = (Don’t know/refused/missing) = missing | Heavy drinking |
| _RFDRHV8 | 2022-2023 | Heavy drinkers (adult men having more than 14 drinks per week and adult women having more than 7  drinks per week) | 1 = No  2 = Yes  9 (Don’t know/refused/missing) = missing | Heavy drinking |
| CHCOCNC1 | 2022-2024 | (Ever told) (you had) melanoma or any other types of  cancer? | 1 = Yes  2 = No  7, 9, or blank (Don’t know/Not sure/Refused/Blank) = Missing | Reported cancer history |
| CHCOCNCR | 2016-2021 | (Ever told) you had any other types of cancer? | 1 = Yes  2 = No  7, 9, or blank (Don’t know/Not sure/Refused/Blank) = Missing | Reported cancer history (yes/no) |
| CSRVTRT3 | 2022-2024 | Are you currently receiving treatment for cancer? | 1 = Yes  2, 3, 4, 5 = No  7, 9, or blank (Don’t know/Not sure/Refused/Blank) = Missing | Current treatment |
| CNCRTYP2 | 2022-2024 | What kind of cancer is it? | Codes 0 to 32 with 0 being no cancer  77, 99, or blank (Don’t know/Not sure/Refused/Blank) = Missing | Cancer type |
| _AGE_G | 2016-2024 | Six-level imputed age category | 1 = 18 to 24  2 = 25 to 34  3 = 35 to 44  4 = 45 to 54  5 = 55 to 64  6 = 65 and older | Age category |
| SEXVAR | 2019-2024 | Sex of Respondent | 1 = Male  2 = Female | Sex |
| SEX1 | 2018 | What is your sex? or What was your sex at birth? Was it… | 1 = Male  2 = Female | Sex |
| SEX | 2016-2017 | Indicate sex of respondent | 1 = Male  2 = Female | Sex |
| _RACEPRV | 2020, 2021, 2023-2024 | Race/ethnicity categories | 1 = Non-Hispanic White  2 = Non-Hispanic Black  3 = Non-Hispanic American/Alaskan Native  4 = Non-Hispanic Asian  5 = Non-Hispanic Asian Pacific Islander  6,7 = Non-Hispanic Other/Multi  8 = Hispanic  Blank = Missing | Race/ethnicity |
| _RACEPR1 | 2022 | Race/ethnicity categories | 1 = Non-Hispanic White  2 = Non-Hispanic Black  3 = Non-Hispanic American/Alaskan Native  4 = Non-Hispanic Asian  5 = Non-Hispanic Asian Pacific Islander  6 = Non-Hispanic Other/Multi  7 = Hispanic | Race/ethnicity |
| _RACE | 2016-2019 | Race/ethnicity categories | 1 = Non-Hispanic White  2 = Non-Hispanic Black  3 = Non-Hispanic American/Alaskan Native  4 = Non-Hispanic Asian  5 = Non-Hispanic Asian Pacific Islander  6, 7 = Non-Hispanic Other/Multi  8 = Hispanic  9 or blank (Don’t know/Not sure/Refused/Blank) = Missing | Race/ethnicity |
| _RFSMOK3 | 2016-2024 | Adults who are current smokers | 1 = No  2 = Yes  9 or blank (Don’t know/Not sure/Refused/Blank) = Missing | Current smoking |
| EDUCA | 2016-2024 | What is the highest grade or year of school you completed? | 1, 2, 3 = < High school  4 = High school or GED  5 = Some college  6 = College or more  9 or blank (Refused/Blank) = Missing | Education |
| _HLTHPL2 | 2024 | Adults who had some form of health insurance | 1 = Insurance  2 = No insurance  9 or blank (Don’t know/Not sure/Refused/Blank) = Missing | Insurance |
| _HLTHPL1 | 2023 | Adults who had some form of health insurance | 1 = Insurance  2 = No insurance  9 or blank (Don’t know/Not sure/Refused/Blank) = Missing | Insurance |
| _HLTHPLN | 2021-2022 | Adults who had some form of health insurance | 1 = Insurance  2 = No insurance  9 or blank (Don’t know/Not sure/Refused/Blank) = Missing | Insurance |
| HLTHPLN1 | 2016-2020 | Do you have any kind of health care coverage, including health insurance, prepaid plans such as HMOs, or government plans such as Medicare, or Indian Health Service? | 1 = Yes  2 = No  9 or blank (Don’t know/Not sure/Refused/Blank) = Missing | Insurance |
| MARITAL | 2016-2024 | Are you: (marital status) | 1 = Married  2, 3, 4, 5, 6 = Unmarried  9 or blank = Missing | Marital status |
| _INCOMG1 | 2021-2024 | Income categories | 1, 2, 3, 4 = <50K  5, 6, 7 = 50K+  9 or blank (Don’t know/Not sure/Refused/Blank) = Missing | Income |
| _INCOMG | 2016-2020 | Income categories | 1, 2, 3, 4 = <50K  5 = 50K+  9 or blank (Don’t know/Not sure/Refused/Blank) = Missing | Income |
| PERSDOC3 | 2021-2024 | Do you have one person or a group of doctors that you  think of as your personal health care provider? | 1, 2 = Yes  3 = No  7, 9, or blank (Don’t know/Not sure/Refused/Blank) = Missing | Personal healthcare provider |
| PERSDOC2 | 2016-2020 | Do you have one person you think of as your personal  doctor or health care provider? | 1, 2 = Yes  3 = No  7, 9, or blank (Don’t know/Not sure/Refused/Blank) = Missing | Personal healthcare provider |

**Supplementary Table 3.** Missing data for variables included in models in 2016–2024 and 2022–2024.

|  | **Trend analyses**  **(2016 to 2024)** | | **Prevalence difference analyses**  **(2022 to 2024)** | |
| --- | --- | --- | --- | --- |
| **Variable** | **No. missing** | **Percent missing** | **No. missing** | **Percent**  **missing** |
| Income | 756848 | 19.1 | 97220 | 20.9 |
| Current drinking | 272928 | 6.9 | 44361 | 9.5 |
| Binge drinking | 311283 | 7.8 | 48608 | 10.4 |
| Heavy drinking | 309115 | 7.8 | 47530 | 10.2 |
| Current smoking | 210912 | 5.3 | 33556 | 7.2 |
| Insurance | 82281 | 2.1 | 19240 | 4.1 |
| Race/ethnicity | 35295 | 0.9 | 16 | 0 |
| Marital status | 34941 | 0.9 | 4522 | 1 |
| Personal healthcare provider | 27444 | 0.7 | 4412 | 0.9 |
| Education | 18373 | 0.5 | 2421 | 0.5 |
| Reported cancer history | 14447 | 0.4 | 2495 | 0.5 |
| Current treatment | NA | NA | 433598 | 93.1 |
| Cancer type | NA | NA | 9265 | 2 |
| Sex | 1463 | 0 | 0 | 0 |
| Age category | 0 | 0 | 0 | 0 |

**Supplementary Table 4.** Distribution of cancer types in BRFSS participants in 2022 and 2024 based on imputed data.

|  | **Including those who reported being in current treatment** | | **Excluding those who reported being in current treatment** | |
| --- | --- | --- | --- | --- |
| **Cancer Type** | **Mean N (Range)** | **Mean % of Total Cancers (Range)** | **Mean N (Range)** | **Mean % of Total Cancers (Range)** |
| None | 412316 (412268-412343) | NA | 411657 (411608-411678) | NA |
| Bladder | 1198 (1195-1201) | 2.2% (2.2-2.2) | 1066 (1061-1075) | 2.1% (2.1-2.2) |
| Bone | 222 (215-237) | 0.4% (0.4-0.4) | 172 (164-186) | 0.3% (0.3-0.4) |
| Brain | 215 (212-220) | 0.4% (0.4-0.4) | 182 (178-189) | 0.4% (0.4-0.4) |
| Breast | 8467 (8430-8511) | 15.8% (15.7-15.9) | 7542 (7487-7614) | 15.2% (15.1-15.3) |
| Cervix/Cervical | 1378 (1374-1381) | 2.6% (2.6-2.6) | 1343 (1339-1347) | 2.7% (2.7-2.7) |
| Colon and rectal | 2184 (2174-2201) | 4.1% (4.1-4.1) | 2037 (2025-2055) | 4.1% (4.1-4.1) |
| Don’t know/Not sure/Refused | 1333 (1311-1353) | 2.5% (2.4-2.5) | 1221 (1202-1245) | 2.5% (2.4-2.5) |
| Esophagus/Esophageal | 195 (191-202) | 0.4% (0.4-0.4) | 166 (162-172) | 0.3% (0.3-0.3) |
| Hematologic | 2367 (2359-2379) | 4.4% (4.4-4.4) | 2060 (2053-2069) | 4.1% (4.1-4.2) |
| Kidney | 1025 (1009-1037) | 1.9% (1.9-1.9) | 959 (944-971) | 1.9% (1.9-2) |
| Liver | 220 (216-226) | 0.4% (0.4-0.4) | 173 (168-178) | 0.3% (0.3-0.4) |
| Lung | 1462 (1429-1509) | 2.7% (2.7-2.8) | 1257 (1222-1309) | 2.5% (2.5-2.6) |
| Melanoma | 6883 (6865-6909) | 12.9% (12.8-12.9) | 6608 (6589-6639) | 13.3% (13.3-13.4) |
| Mouth/tongue/lip | 162 (156-179) | 0.3% (0.3-0.3) | 154 (148-170) | 0.3% (0.3-0.3) |
| Other | 9342 (9297-9410) | 17.4% (17.4-17.6) | 8952 (8890-8994) | 18% (18-18.1) |
| Ovary/Ovarian | 626 (620-632) | 1.2% (1.2-1.2) | 576 (568-584) | 1.2% (1.1-1.2) |
| Pancreas/Pancreatic | 242 (241-244) | 0.5% (0.4-0.5) | 200 (197-202) | 0.4% (0.4-0.4) |
| Prostate | 5290 (5260-5337) | 9.9% (9.8-10) | 4748 (4715-4791) | 9.6% (9.5-9.6) |
| Skin^a^ | 7685 (7643-7726) | 14.4% (14.3-14.4) | 7322 (7290-7359) | 14.7% (14.7-14.8) |
| Stomach | 172 (169-178) | 0.3% (0.3-0.3) | 148 (144-154) | 0.3% (0.3-0.3) |
| Testis/Testicular | 272 (271-273) | 0.5% (0.5-0.5) | 263 (261-265) | 0.5% (0.5-0.5) |
| Throat - pharynx | 268 (263-274) | 0.5% (0.5-0.5) | 255 (249-260) | 0.5% (0.5-0.5) |
| Thyroid | 1210 (1200-1219) | 2.3% (2.2-2.3) | 1147 (1138-1152) | 2.3% (2.3-2.3) |
| Uterus/Uterine | 1136 (1128-1147) | 2.1% (2.1-2.1) | 1097 (1084-1115) | 2.2% (2.2-2.2) |

^a^Includes reported non-melanoma skin cancer and types where the participant reported they did not know what kind of skin cancer was diagnosed.

**Supplementary Table 5.** Weighted and unweighted prevalences of current drinking, binge drinking, and heavy drinking.

|  |  | **Current drinking** | | **Binge drinking** | | **Heavy drinking** | |
| --- | --- | --- | --- | --- | --- | --- | --- |
| **Reported**  **cancer?** | **Year** | **Unweighted**  **Prevalence (%)**  **(95% CI)** | **Weighted**  **Prevalence (%)**  **(95% CI)** | **Unweighted**  **Prevalence (%)**  **(95% CI)** | **Weighted**  **Prevalence (%)**  **(95% CI)** | **Unweighted**  **Prevalence (%)**  **(95% CI)** | **Weighted**  **Prevalence (%)**  **(95% CI)** |
| No | 2016 | 52.1 (52.1–52.2) | 53.7 (53.4–54) | 13.9 (13.9–14) | 17.7 (17.4–17.9) | 6.1 (6.1–6.2) | 6.5 (6.4–6.7) |
| Yes | 2016 | 44.5 (44.2–44.8) | 45.7 (44.7–46.8) | 6.6 (6.5–6.7) | 8.5 (7.8–9.1) | 5 (4.9–5.1) | 5.2 (4.7–5.7) |
| No | 2017 | 52.7 (52.6–52.8) | 54.1 (53.7–54.5) | 14.3 (14.2–14.4) | 17.8 (17.5–18.1) | 6 (6–6.1) | 6.3 (6.1–6.5) |
| Yes | 2017 | 45.7 (45.4–45.9) | 46.8 (45.7–47.8) | 6.9 (6.8–7) | 9.1 (8.3–9.8) | 4.9 (4.7–5) | 5.4 (4.9–5.8) |
| No | 2018 | 52.3 (52.2–52.4) | 53.1 (52.7–53.4) | 14.4 (14.4–14.5) | 17.2 (17–17.5) | 6.3 (6.3–6.4) | 6.5 (6.3–6.7) |
| Yes | 2018 | 44.6 (44.3–44.8) | 44.7 (43.6–45.8) | 6.8 (6.7–6.9) | 8.1 (7.5–8.7) | 4.9 (4.8–5) | 5.1 (4.7–5.6) |
| No | 2019 | 51.2 (51.1–51.3) | 53 (52.6–53.4) | 14.4 (14.3–14.4) | 17.6 (17.3–17.9) | 6.2 (6.2–6.3) | 6.6 (6.4–6.7) |
| Yes | 2019 | 43.8 (43.5–44) | 45 (44–46) | 7 (6.9–7.2) | 9.1 (8.4–9.8) | 4.9 (4.8–5) | 5.6 (5–6.2) |
| No | 2020 | 51.7 (51.6–51.7) | 52.1 (51.7–52.5) | 14.3 (14.2–14.4) | 16.3 (16–16.6) | 6.7 (6.7–6.8) | 6.8 (6.6–7) |
| Yes | 2020 | 44.1 (43.9–44.4) | 44.5 (43.3–45.8) | 6.8 (6.6–6.9) | 8 (7.4–8.6) | 5.3 (5.1–5.4) | 5.6 (5.1–6.2) |
| No | 2021 | 52.4 (52.4–52.5) | 52.6 (52.3–53) | 14.3 (14.3–14.4) | 16.4 (16.1–16.7) | 6.2 (6.1–6.2) | 6.2 (6–6.4) |
| Yes | 2021 | 44.6 (44.3–44.9) | 43.6 (42.5–44.7) | 6.9 (6.7–7) | 8.1 (7.5–8.7) | 4.8 (4.6–4.9) | 4.8 (4.3–5.2) |
| No | 2022 | 53.3 (53.2–53.4) | 52.7 (52.3–53.1) | 15.6 (15.5–15.7) | 17.5 (17.3–17.8) | 6.9 (6.8–6.9) | 6.8 (6.6–7) |
| Yes | 2022 | 48.6 (48.3–48.8) | 47 (46–48) | 8.1 (7.9–8.2) | 9.4 (8.7–10.1) | 5.7 (5.5–5.8) | 6 (5.5–6.5) |
| No | 2023 | 53.1 (53–53.2) | 51.9 (51.6–52.3) | 14.4 (14.3–14.4) | 16.1 (15.8–16.4) | 6.1 (6.1–6.2) | 5.9 (5.7–6.1) |
| Yes | 2023 | 48.8 (48.6–49.1) | 47.2 (46.2–48.3) | 7.1 (7–7.2) | 8.4 (7.7–9.1) | 5.1 (5–5.2) | 5.1 (4.7–5.6) |
| No | 2024 | 51.2 (51.1–51.3) | 51.2 (50.9–51.6) | 14.5 (14.4–14.5) | 16.3 (16–16.5) | 6.1 (6–6.1) | 5.8 (5.6–5.9) |
| Yes | 2024 | 46.5 (46.3–46.8) | 45.6 (44.7–46.6) | 7.3 (7.2–7.4) | 9 (8.4–9.6) | 5.1 (5–5.2) | 5.4 (5–5.9) |

**Supplementary Table 6.** Linear regression model results for trend analyses.

| **APC** | **p-value** | **Lower 95% CI** | **Upper 95% CI** | **Model** |
| --- | --- | --- | --- | --- |
| -0.29667 | 0.000835 | -0.42244 | -0.17089 | No reported cancer/current drinking |
| 0.066667 | 0.705868 | -0.33425 | 0.467581 | Reported cancer/current drinking |
| -0.18833 | 0.023778 | -0.34317 | -0.0335 | No reported cancer/binge drinking |
| 0.025 | 0.737778 | -0.14467 | 0.194674 | Reported cancer/binge drinking |
| -0.06333 | 0.188783 | -0.1662 | 0.039537 | No reported cancer/heavy drinking |
| 0.015 | 0.766201 | -0.09973 | 0.129729 | Reported cancer/heavy drinking |

**Supplementary Table 7.** Weighted adjusted prevalence difference estimates for current drinking by cancer type (reference = No cancer history) including and excluding those with a cancer history who reported being in current treatment.

|  | **Including those in current treatment** | **Excluding those in current treatment^a^** | **Closer to the null?** |
| --- | --- | --- | --- |
| **Cancer site** | **PD (%) (95% CI)^b^** | **PD (%) (95% CI)^b^** | Yes |
| Bladder | -1.9 (-2.99 to -0.8) | -0.79 (-1.89 to 0.31) | No |
| Bone | -7.94 (-13.4 to -2.48) | -9.92 (-16.62 to -3.21) | No |
| Brain | -8.05 (-9.03 to -7.07) | -9.61 (-10.59 to -8.62) | Yes |
| Breast | -1.01 (-1.48 to -0.55) | -0.76 (-1.25 to -0.27) | Yes |
| Cervix/Cervical | -3 (-4.38 to -1.62) | -2.95 (-4.37 to -1.54) | Yes |
| Colon and rectal | -3.61 (-4.96 to -2.26) | -2.74 (-4.13 to -1.34) | Yes |
| Don’t know/Not sure/Refused | -2.4 (-3.91 to -0.9) | -2.11 (-3.78 to -0.45) | Yes |
| Esophagus/Esophageal | -9.54 (-10.94 to -8.15) | -6.03 (-7.6 to -4.47) | Yes |
| Hematologic | -4 (-4.53 to -3.48) | -2.9 (-3.43 to -2.36) | Yes |
| Kidney | -8.19 (-9.49 to -6.89) | -7.11 (-8.4 to -5.83) | Yes |
| Liver | -18.64 (-21.61 to -15.67) | -14.84 (-18.55 to -11.13) | Yes |
| Lung | -9.16 (-9.93 to -8.39) | -8.92 (-10 to -7.83) | Yes |
| Melanoma | 2.58 (2.26 to 2.91) | 2.54 (2.16 to 2.92) | Yes |
| Mouth/tongue/lip | -7.86 (-10.8 to -4.92) | -6.88 (-10.03 to -3.72) | Yes |
| Other | -1.57 (-3.06 to -0.09) | -1.28 (-2.81 to 0.25) | Yes |
| Ovary/Ovarian | -5.8 (-7.37 to -4.23) | -3.99 (-5.8 to -2.19) | Yes |
| Pancreas/Pancreatic | -15.36 (-16.86 to -13.85) | -13.32 (-15.69 to -10.95) | No |
| Prostate | 1.41 (1.08 to 1.75) | 2.06 (1.77 to 2.36) | No |
| Skin^c^ | 3.86 (3.39 to 4.32) | 4.09 (3.6 to 4.57) | Yes |
| Stomach | -5.39 (-9.6 to -1.18) | -2.53 (-7.42 to 2.36) | Yes |
| Testis/Testicular | 10.33 (8.37 to 12.29) | 10.17 (8.13 to 12.2) | No |
| Throat - pharynx | 0.33 (-1.24 to 1.89) | 1.14 (-0.64 to 2.92) | Yes |
| Thyroid | -5.12 (-6.64 to -3.61) | -4.71 (-6.27 to -3.16) | No |
| Uterus/Uterine | -6.08 (-6.91 to -5.24) | -6.61 (-7.32 to -5.9) | Yes |

^a^n = 4,160 excluded who reported being in current treatment in the parent dataframe for the imputation (no imputed values included)

^b^Adjusted for Models were adjusted for age category, race ethnicity category, insurance category, education category, smoking category, marital category, income category, and personal healthcare provider.

^c^Includes reported non-melanoma skin cancer and types where the participant reported they did not know what kind of skin cancer was diagnosed.

**Supplementary Figure 1.** Unweighted prevalences by reported cancer history of (**A**) current drinking, (**B**) heavy drinking, and (**C**) binge drinking.
